# Probability-based collaborative filtering model for predicting gene–disease associations

**DOI:** 10.1186/s12920-017-0313-y

**Published:** 2017-12-28

**Authors:** Xiangxiang Zeng, Ningxiang Ding, Alfonso Rodríguez-Patón, Quan Zou

**Affiliations:** 1 Department of Computer Science, School of information science and technology, Xiamen University, Xiamen, China; 2 Department of Artificial Intelligence, Universidad Politcnica de Madrid (UPM), Madrid, Spain; 3 School of Computer Science and Technology, Tianjin University, Tianjin, China

**Keywords:** Gene–disease association prediction, Latent factor model, Heterogeneous similarity regularization, Biological network

## Abstract

**Background:**

Accurately predicting pathogenic human genes has been challenging in recent research. Considering extensive gene–disease data verified by biological experiments, we can apply computational methods to perform accurate predictions with reduced time and expenses.

**Methods:**

We propose a probability-based collaborative filtering model (PCFM) to predict pathogenic human genes. Several kinds of data sets, containing data of humans and data of other nonhuman species, are integrated in our model. Firstly, on the basis of a typical latent factorization model, we propose model I with an average heterogeneous regularization. Secondly, we develop modified model II with personal heterogeneous regularization to enhance the accuracy of aforementioned models. In this model, vector space similarity or Pearson correlation coefficient metrics and data on related species are also used.

**Results:**

We compared the results of PCFM with the results of four state-of-arts approaches. The results show that PCFM performs better than other advanced approaches.

**Conclusions:**

PCFM model can be leveraged for predictions of disease genes, especially for new human genes or diseases with no known relationships.

## Background

It is a material trial in biology that correctly predicting novel pathogenic genes associated with human diseases. However, detecting gene–disease relationships can be challenging [1].

Many strategies have been proposed to predict gene–disease associations. In general, a prediction method is based on “guilt by association” (GBA) principle [2]. In this principle, novel pathogenic genes are determined on the basis of the associations between such genes and relevant neighboring genes. Approaches integrating diverse data sources have been generally exploited for predictions of pathogenic human genes. For instance, CIPHER [3], GeneWalker [4], Prince [5], RWRH [6], Katz and CATAPULT [7], inductive matrix completion [8]. A number of varieties of evidence continually exploited for prediction of gene–disease ralationships were studied by Piro and Di Cunto [9]. With a text-mining method Driel et al. detected the relationships of human genes related to diseases determined in the Online Mendelian Inheritance in Man (OMIM) [10, 11]. In protein interaction data, Köhler et al. predicted pathogenic human genes using random walk to regulate similarities. The random walk is verified to be more precise than other methods [12]. For analyzing protein interaction, an approach “network propagation” has also been developed on the basis of random walk [13, 14].

The predictions of gene–disease relationships can be considered as designing a recommender system to commend the items (genes) of interest to a user (disease) on the basis of the preference that a gene possibly encodes a disease. Users related with one another in recommender systems likely experience mutual tastes or share similar interests in accordance with homophily principle [15]. Recommender systems usually rely on collaborative filtering (CF) [16], which depends on prior disposals to predict relationships between users and items. CF has been widely applied effectively in many practices [17]. CF has also been adopted in some remarkable advancements by some renowned companies, including Amazon [18], TiVo, and Netflix, because of the simplicity and effectiveness of this technique. In CF, users A and B similarly act on or rate other items if these users demonstrate a similar behavior or likewise rate *n* items [19]. The two main methods of CF are latent factor models and neighborhood models. In latent factor models, evidence of both users and items is integrated. In neighborhood models, similarities between users and items are examined. Regarded as optimum methods to obtain more accurate consequences in Netflix prize, latent factor models, such as matrix factorization, have been widely used in recommender systems [20]. Recommender systems have also been used in other models [21–24]. Koren et al. suggested a combined model with high prediction accuracy by leveraging the superiorities of both neighborhood and latent factor methods. Recent studies [23, 24] integrated a network-based similarity property between users into advanced matrix factorization recommendation approaches [25].

In this study, we proposed a probability-based collaborative filtering model (PCFM) for prediction of gene–disease relationships. As neighborhood models can not cover overall demonstrated information, latent factor models were chosen for our proposed model. On the basis of traditional latent factor models, we defined an additional probability-based approach which can detect unknown relationships. The prediction of gene–disease associations has been considered a semi-supervised learning problem because of few certified relationships. In this study, this semi-supervised learning problem was translated into an acquainted supervised learning problem with PCFM. If values in gene–disease association matrix are 0 or 1, predictions are regarded as binary classification problems. The collaborative filtering approach leveraged in the recommender system was designed to rate matrix with precise scores. Hence, the models cannot be immediately exploited in the predictions for gene–disease associations. Two models with regularization were developed to modify the basic model.

## Methods

### Datasets

**Fig. 1:**
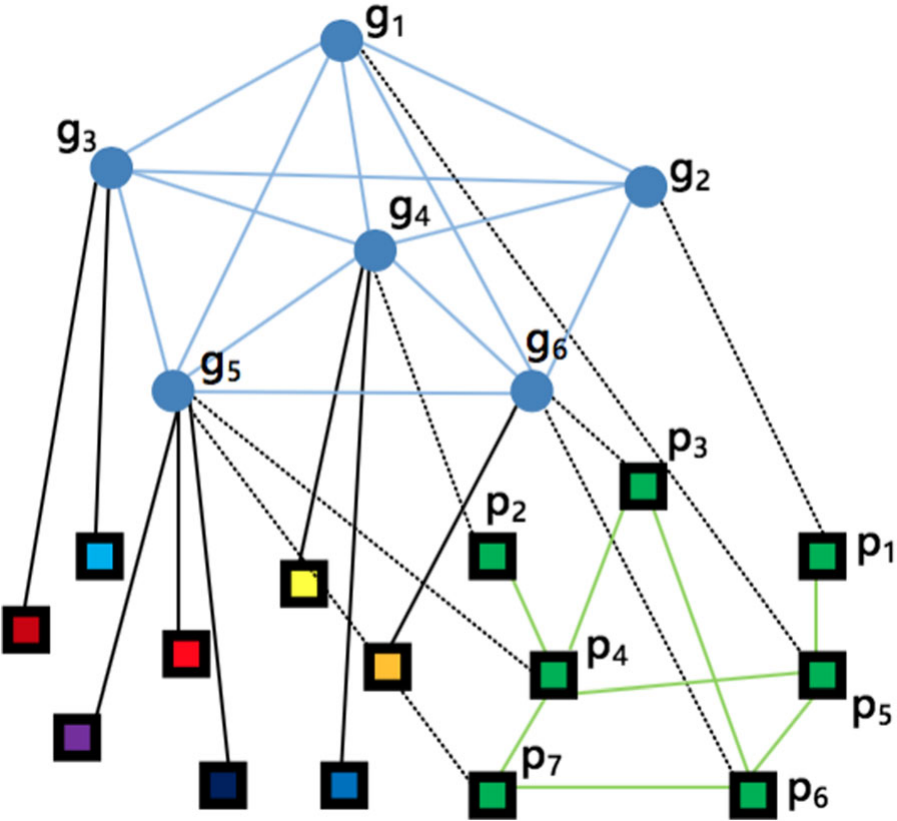
Heterogeneous network of genes and diseases

Three types of data sets are shown in Fig. 1. We obtained gene–gene relationships from HumanNet [26], which includes 12,331 human genes. HumanNet with 733,836 linkages is a genome-scale human genes network, constructed based on 21 diverse proteomics and genomics evidences, including four species: protein–protein interactions, human mRNA coexpression, comparative genomics data sets, and protein complex data sets. Different data sets were fused into a functional gene-gene relationship network.

The gene–disease relationships were downloaded from Online Mendelian Inheritance in Man (OMMI), the standard dataset to appraise predictions of pathogenic human genes. OMIM is a authoritative and comprehensive compendium of human genes and genetic diseases which is updated daily and freely available on the website. The full-text, referenced overviews in OMIM contain information on all known mendelian disorders. This standard dataset contains numerous linkages with other genetics resources. OMIM has been developing since it was initiated in the early 1960s, while it was made generally available on the internet starting in 1987. Nowadays, OMIM was developed for the World Wide Web by the National Center for Biotechnology Information (NCBI). With 3209 diseases connecting at least one known gene and 3954 demonstrated linkages, this network is frequently leveraged in researches of genes. In our experiment, given the existence of orthologous genes in humans and other species [27], we append the gene-disease linkages between orthologous genes and eight nonhuman species diseases. The description of associations between orthologous genes and diseases of eight other nonhuman species can be found in [28].

And the disease–disease similarity associations was downloaded from [28]. This dataset provides similarities between human diseases, which show pertinence between genes with similar interactive function. The disease-disease association network has a positive influence on prediction for gene–disease associations. This network contains 3209 diseases and 3,165,225 entries. In this network, values of edges measure the degree of similarity.

### Latent factor models

Recommender systems involve various input data, including items and users, which often correspond to a matrix. In a rating matrix *R* ^m × *n*^, *m* represents the number of users, and *n* represents the number of items. The basic latent factor models manage to characterize users and items by using vectors of factors inferred from item-rating patterns. A high accordance exists between user and item factor issues in a recommendation. Latent factor models map items and users to a corporate latent factor space of dimensionality *D*, in which users are related to matrix *U* ∈ ℝ^*m* × *D*^, and items are related to matrix *V* ∈ ℝ^*n* × *D*^. The approximate rating matrix acquires the overall interest of users in the characteristics of items and is denoted by

1 $$ R\approx {UV}^T $$

where *U* represents the training feature matrix of users in the latent factor space, in which the *i*th row corresponds to the user feature vector *u*_*i*_; and *V* represents the training feature matrix of items, in which the *i*th row corresponds to the item feature vector *v*_*i*_.

The user–item matrix is a very sparse matrix with a large number of undiscovered scores in general. To avoid insignificant calculations, the least square optimization algorithm is exploited for recommender systems to settle the problem, and the specific optimization equation is

2 $$ {\mathit{\min}}_{U,V}\frac{1}{2}{\sum}_{i=1}^m{\sum}_{j=1}^n{I}_{ij}{\left({R}_{ij}-{U}_i{V}_j^T\right)}^2+\frac{\lambda_1}{2}{\left\Vert U\right\Vert}^2+\frac{\lambda_2}{2}{\left\Vert V\right\Vert}^2 $$

where *λ*_1_, *λ*_2_ > 0, and *I*_*ij*_ is an indicator function, for which if *R*_*ij*_ is known, *I*_*ij*_ = 1, otherwise, *I*_*ij*_ = 0. Two different approaches are leveraged ordinarily to minimize the objective function: alternating least squares method and stochastic gradient descent method. Alternating least squares rotate by calculating the partial derivatives for factor vectors of users and items, and then the method sets them both equal to zero. Multiple repetitions of this process assure that each step decreases the equation until convergence [29]. The stochastic gradient descent method randomly defines an initial value and calculates the related prediction error. The parameters are then modified in the opposite direction of the gradient. This method is popular and successful in many cases [21, 30, 31]. Although the alternating least squares method is favorable in systems using parallelization [32] and systems centered on implicit data [33], this method is generally more complex and slower than the stochastic gradient descent. Therefore, the latter is exploited in this study.

### Semi-supervised learning method

The main difficulty of predicting gene–disease associations can be ascribed to the lack of negative samples in the training process. For the imperfection of gene–disease data, we can obtain two pivotal specialties of our experimental data: (1) for every disease, few known genes are related to it; we may know the relevant genes for the disease, but we are unaware of the irrelevant ones; (2) many unlabeled gene–disease pairs exist with the prior information, but most of them are negative associations [7].

We can utilize the semi-supervised learning method for our experiments. Liu et al. searched different varieties of approaches to select negative samples [34]. Given that recent studies showed few positive samples in the gene–disease dataset, if we select a sample from the gene–disease matrix, the sample is likely to be a positive one; as a consequence, negative samples can be selected randomly from the training dataset. Mordelet et al. proposed a ProDiGe method to select negative samples by adopting the PU learning framework [35].

In our experiment, we chose the random walk method to select negative samples. If *P*_*gd*_ denotes the probability that gene *g* walks to human disease *d*, *P*_*gd*_ is formulated by

3 $$ {P}_{gd}={S}_g{I}_{GD}{S}_d/\left(\left|{S}_g\right|\left|{S}_d\right|\right) $$

where *S*_*g*_ and *S*_*d*_ represent the human gene similarity matrix and disease similarity matrix, respectively, and *I*_*GD*_ is an adjacent matrix showing the known associations between human genes and diseases in OMMI. If *NS* denotes a negative sample set, and *θ* is assigned to be the threshold value, then *NS* = {*NS*_≤*θ*_, *NS*_>*θ*_}. The negative samples indicating *NS*_≤*θ*_ are selected from the samples with probabilities are less than *θ* in *P*_*gd*_, and *NS*_>*θ*_ is selected from the samples with probabilities of more than *θ* in *P*_*gd*_; the number of *NS*_>*θ*_ negative samples is small. We choose negative samples with probabilities of more than *θ* in *P*_*gd*_ because as the prior information shows, most samples in the gene–disease association matrix are negative samples; therefore, to enhance the generalizability of this model, few *NS*_>*θ*_ negative samples are joined.

### Basic model: probability-based collaborative filtering model

In recommender systems, the latent factor models designing for the rating matrix with precise scores cannot be used in the gene–disease association matrix with binary scores of 0 or 1. Similarly, alternating least squares cannot work in this experiment.

**Fig. 2:**
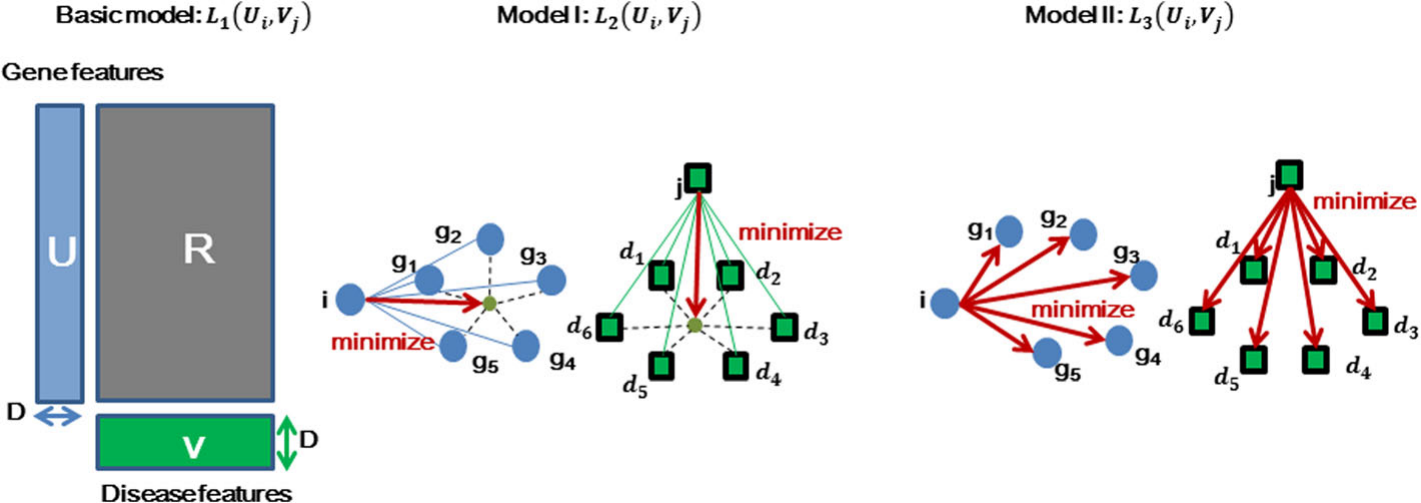
Description of three models

The basic model is shown in Fig. 2. Let *P*(*Y* = 1|*U*_*i*_, *V*_*j*_) denote the probability that human gene *i* is related to disease j, and let *P*(*Y* = 0|*U*_*i*_, *V*_*j*_) denote the probability that this gene is irrelevant to that disease. We define matrix *U* ∈ ℝ^*m* × *D*^ and *V* ∈ ℝ^*n* × *D*^ as the feature matrixes of human genes and diseases in the latent factor space of dimensionality *D*. We define *P*(*Y* = 1|*U*_*i*_, *V*_*j*_) and *P*(*Y* = 0|*U*_*i*_, *V*_*j*_) as

4 $$ P\left({Y}_{ij}=1\left|{U}_i,{V}_j\right.\right)=\frac{1}{\mathit{\exp}\left(f\left({U}_i,{V}_j\right)\right)} $$

5 $$ P\left({Y}_{ij}=0\left|{U}_i,{V}_j\right.\right)=\frac{\mathit{\exp}\left(f\left({U}_i,{V}_j\right)\right)-1}{\mathit{\exp}\left(f\left({U}_i,{V}_j\right)\right)} $$

In Eq. (5), $f\left({U}_i,{V}_j\right)=\frac{{\left\Vert {U}_i-{V}_j^T\right\Vert}^2}{2}+\vartheta$, and *ϑ* is a tiny positive number; in this experiment, *ϑ* is set as 0.0001. We can see that, if *f*(*U*_*i*_, *V*_*j*_) → 0,  *P*(*Y*_*ij*_ = 1|*U*_*i*_, *V*_*j*_) → 1; if *f*(*U*_*i*_, *V*_*j*_) →  ∞ ,  *P*(*Y*_*ij*_ = 0|*U*_*i*_, *V*_*j*_) → 1.

*U*_*i*_ and *V*_*j*_ is obtained by using the maximum likelihood estimate. We define that *P*(*Y*_*ij*_ = 1|*U*_*i*_, *V*_*j*_) = *π*(*U*_*i*_, *V*_*j*_) and *P*(*Y*_*ij*_ = 0|*U*_*i*_, *V*_*j*_) = 1 − *π*(*U*_*i*_, *V*_*j*_), the likelihood function is

6 $$ {\prod}_{i,j}^{m,n}{\left[\pi \left({U}_i,{V}_j\right)\right]}^{Y_{ij}}{\left[1-\pi \left({U}_i,{V}_j\right)\right]}^{1-{Y}_{ij}} $$

The log-likelihood function is

7 $$ {\displaystyle \begin{array}{l}{L}_1\left({U}_i,{V}_j\right)={\sum}_{i,\kern0.5em j}^{m,\kern0.5em n}\left[{Y}_{ij}\kern0.5em \mathit{\log}\kern0.5em \pi \left({U}_i,{V}_j\right)+\left(1-{Y}_{ij}\right)\mathit{\log}\left(1-\pi \left({U}_i,{V}_j\right)\right)\right]\\ {}={\sum}_{i,\kern0.5em j}^{m,\kern0.5em n}\left[\left(1-{Y}_{ij}\right)\mathit{\log}\left(1-{e}^{-f\left({U}_i,{V}_j\right)}\right)-{Y}_{ij}f\left({U}_i,{V}_j\right)\right]\end{array}} $$

The maximum value of *L*_1_(*U*_*i*_, *V*_*j*_) is calculated, and then we obtain the estimated value of *L*_1_(*U*_*i*_, *V*_*j*_). Subsequently, the stochastic gradient descent method is used to solve this problem. The formulas updating the gradients of *U*_*i*_ and *V*_*j*_ are

8 $$ \frac{\partial {L}_1}{\partial {U}_i}={\sum}_j^n\left({V}_j^T-{U}_i\right)\left[{Y}_{ij}+\left(1-{Y}_{ij}\right)\left(1-\frac{1}{\left(1-{e}^{-f\left({U}_i,{V}_j\right)}\right)}\right)\right] $$

9 $$ \frac{\partial {L}_1}{\partial {V}_i}={\sum}_i^m\left({U}_i-{V}_j^T\right)\left[{Y}_{ij}+\left(1-{Y}_{ij}\right)\left(1-\frac{1}{\left(1-{e}^{-f\left({U}_i,{V}_j\right)}\right)}\right)\right] $$

### Computation of gene–gene similarities

We utilize the relationships between orthologous genes and diseases of nonhuman species to calculate the gene–gene similarities. Vector space similarity (VSS) and Pearson correlation coefficient (PCC) [36] is exploited to evaluate the gene–gene similarities. The formula of VSS is

10 $$ {S}_{ig}=\frac{\sum_{j\in I(i)\cap I(g)}\kern0.5em {R}_{ij}\cdot {R}_{gj}}{\sqrt{\sum_{j\in I(i)\cap I(g)}{R}_{ij}^2}\cdot \sqrt{\sum_{j\in I(i)\cap I(g)}{R}_{f\kern0em j}^2}} $$

*I*(*g*)Z denote the diseases of eight other species related to orthologous gene g, let *I*(*i*) represent the diseases of eight other species related to orthologous gene *i*, and we define *j* as the vertexes that *I*(*i*) and *I*(*g*) have in common. *S*_*ig*_ ranges from 0 to 1, measuring the level of similarity between gene *i* and gene g. *R*_*ij*_ is defined as the linkage between gene *i* and disease *j* of other nonhuman species, where value 1 shows correlation and value 0 shows irrelevance. The definition of *S*_*ig*_ is ${S}_{ig}=\frac{\left|I(i)\right|}{{\mathit{\max}}_{j\in m}\left|I(j)\right|}\cdot {S}_{ig}$.

However, in VSS, some genes in *I*(*i*) and *I*(*g*) which make a larger contribution to predictions are taken into consideration. Thus, a formula using PCC is defined to deal with this deficiency:

11 $$ {S}_{ig}=\frac{\sum_{j\in I(i)\cap I(g)}\left({R}_{ij}-\overline{R_i}\right)\cdot \left({R}_{gj}-\overline{R_g}\right)}{\sqrt{\sum_{j\in I(i)\cap I(g)}{\left({R}_{ij}-\overline{R_i}\right)}^2}\cdot \sqrt{\sum_{i\in I(i)\cap I(g)}{\left({R}_{gj}-\overline{R_g}\right)}^2}} $$

Where ${\overline{R}}_i$ represents the average value of similarities between gene *i* and overall nonhuman diseases. We map the value of PCC to [0,1] by using the mapping function *f*(*x*) = (*x* + 1)/2. Approximately, let the definition of *S*_*ig*_ be ${S}_{ig}=\frac{\left|I(i)\right|}{{\mathit{\max}}_{j\in m}\left|I(j)\right|}\cdot {S}_{ig}$.

Let ${S}_g^{\prime }$ denote similarities between human genes and diseases of eight other species, and we acquire ${S}_g^{{\prime\prime} }$ from HumanNet. With mapping function, we map ${S}_g^{{\prime\prime} }$ to [0,1]. Ultimately, the definition of gene–gene similarities is ${S}_g={S}_g^{\prime }+\omega \cdot normal\left\{{S}_g^{{\prime\prime}}\right\}$, and weight *ω* is determined by the significances of ${S}_g^{\prime }$ and ${S}_g^{{\prime\prime} }$. In our work, *ω* is larger than 1, for HumanNet is widely believed to be more reliable on account of using prior information.

### ModelI: Probability-based collaborative filtering model with integral regularization

We add more prior information in model I, containing gene–gene relationships and disease–disease similarities. The model I is defined as

12 $$ {\mathit{\max}}_{U_i,{V}_j}{L}_2\left({U}_i,{V}_j\right)={L}_1\left({U}_i,{V}_j\right)-\frac{\alpha_1}{2}{\sum}_i^m{\left\Vert {U}_i-\frac{\sum_{g\in G(i)}{S}_{ig}\times {U}_g}{\sum_{g\in G(i)}{S}_{ig}}\right\Vert}^2-\frac{\beta_1}{2}{\sum}_j^n{\left\Vert {V}_j-\frac{\sum_{d\in D(j)}{S}_{jd}\times {V}_d}{\sum_{d\in D(j)}{S}_{jd}}\right\Vert}^2 $$

In this equation, the neighbor genes of gene *i* is denoted as *G*(*i*), the neighbor genes of disease *j* is denoted as *D*(*j*), and *α*_1_, *β*_1_ > 0. *S*_*ig*_ ∈ [0, 1] and *S*_*jd*_ ∈ [0, 1] represents the similarities between human genes and their neighbor genes.

We add two integral regularizations of human genes and diseases in model I:

13 $$ \frac{\alpha_1}{2}{\sum}_i^m{\left\Vert {U}_i-\frac{\sum_{g\in G(i)}{S}_{ig}\times {U}_g}{\sum_{g\in G(i)}{S}_{ig}}\right\Vert}^2 $$

14 $$ \frac{\beta_1}{2}{\sum}_j^n{\left\Vert {V}_j-\frac{\sum_{p\in P(j)}{S}_{jp}\times {V}_p}{\sum_{p\in P(j)}{S}_{jp}}\right\Vert}^2 $$

We can see that in Fig. 2, we should minimize the two regularizations to make *L*_2_(*U*_*i*_, *V*_*j*_) maximal. As such, we should make gene *i* and disease *j* close to the center of the Euclidean distance between gene *i* and its neighbors *G*(*i*), as well as between disease *j* and its neighbors *D*(*j*) and *S*_*jd*_ can be gotten from the published dataset, and the computation of *S*_*ig*_ will be specified hereinbelow.

### Model II: Probability-based collaborative filtering model with personal regularization

However, while there are big differences between similarities of genes and diseases respectively, model I may give a erroneous result.

A Probability-based collaborative filtering model with personal regularization called model II is defined to cope with this circumstance, and we define model II as

15 $$ {\mathit{\max}}_{U_i,{V}_j}{L}_3\left({U}_i,{V}_j\right)={L}_1\left({U}_i,{V}_j\right)-\frac{\alpha_2}{2}{\sum}_i^m{\sum}_{g\in G(i)}{S}_{ig}{\left\Vert {U}_i-{U}_g\right\Vert}^2-\frac{\beta_2}{2}{\sum}_j^n{\sum}_{d\in P(j)}{S}_{dt}{\left\Vert {V}_j-{V}_d\right\Vert}^2 $$

As shown in Fig. 2. *α*_2_, *β*_2_ > 0, and other parameters can be explained similarly Eq. (10).

Model II can adjust the distance between genes or diseases in the latent factor space indirectly. Briefly, if gene *g* is a neighbor of gene *i*, and gene *f* is a neighbor of gene *g* in model II, the distance between *U*_*i*_ and *U*_*f*_ in a latent factor space will be minimized indirectly as follows:

$$ {S}_{ig}{\left\Vert {U}_i-{U}_g\right\Vert}^2,\kern0.5em {S}_{gf}{\left\Vert {U}_g-{U}_f\right\Vert}^2 $$

This formula will finally realize the convergence, reaching a steady status of the transmission process.

And the formulas updating the gradient for the stochastic gradient descent approach are

16 $$ \frac{\partial {L}_3}{\partial {U}_i}={\sum}_j^n\left({V}_j^T-{U}_i\right)\left[{Y}_{ij}+\left(1-{Y}_{ij}\right)\left(1-\frac{1}{\left(1-{e}^{-f\left({U}_i,{V}_j\right)}\right)}\right)\right]-{\alpha}_2{\sum}_{g\in G(i)}{S}_{gt}\left({U}_i-{U}_g\right) $$

17 $$ \frac{\partial {L}_3}{\partial {V}_i}={\sum}_i^m\left({U}_i-{V}_j^T\right)\left[{Y}_{ij}+\left(1-{Y}_{ij}\right)\left(1-\frac{1}{\left(1-{e}^{-f\left({U}_i,{V}_j\right)}\right)}\right)\right]-{\beta}_2{\sum}_{p\in P(j)}{S}_{pt}\left({V}_j-{V}_p\right) $$

## Results and discussion

### Comparing with state-of-arts methods

As a semi-supervised learning problem, prediction for pathogenic genes meets with a “cold start” problem, and we propose PCFM to solve it. For that gene–disease relationship network is very sparse, some human diseases exist with no known associated genes. A threefold cross validation is performed to compare the result of our PCFM approach with other state-of-arts methods: Katz [7] and Catapult [7] that are based on numbers of different paths, Prince [14] that involves global networks, and ProDiGe [35] that integrate numerous biological datasets.

Katz is a graph-based approach for detecting vertexes related to a given one. This method has performed well for recommending human genes for a given diseases. In this method, the similarity between two vertexes depends on the number of walks of different lengths from one vertex to another. The formula of Katz is

18 $$ {S}_{Hs}^{Katz}(C)=\beta {P}_{Hs}+{\beta}^2\left({GP}_{Hs}+{P}_{Hs}{Q}_{Hs}\right)+{\beta}^3\left({PP}^T{P}_{Hs}+{G}^2{P}_{Hs}+{GP}_{Hs}{Q}_{Hs}+{P}_{Hs}{Q}_{Hs}^2\right) $$

In this equation, *P*_*Hs*_ and *Q*_*Hs*_ represent the gene–gene matrix and disease–disease matrix, and *β* is a constant which can punish long walks. In this way, gene–disease score can be calculated by Katz method.

Catapult is a method which can learn different weights for paths of different lengths. It try to find out a score for each gene–disease pair, which can be treated as learning coefficients for Katz. As a result of lack of known negative examples, Positive-Unlabeled learning (PU learning) approaches is utilized in this method, to establish a negative set, and gene–disease pairs are classified leveraging a biased support vector machine.

ProDiGe is a novel approach based on support vector machine. It learns from positive and unlabeled examples. In order to get more precise prediction, 21 diverse evidences of genes and diseases were used for computing gene–gene similarities.

Prince is a comprehensive approach which based on usage of prior information and formulating constraints on the prioritization function that relate to its smoothness over the network.

**Fig. 3:**
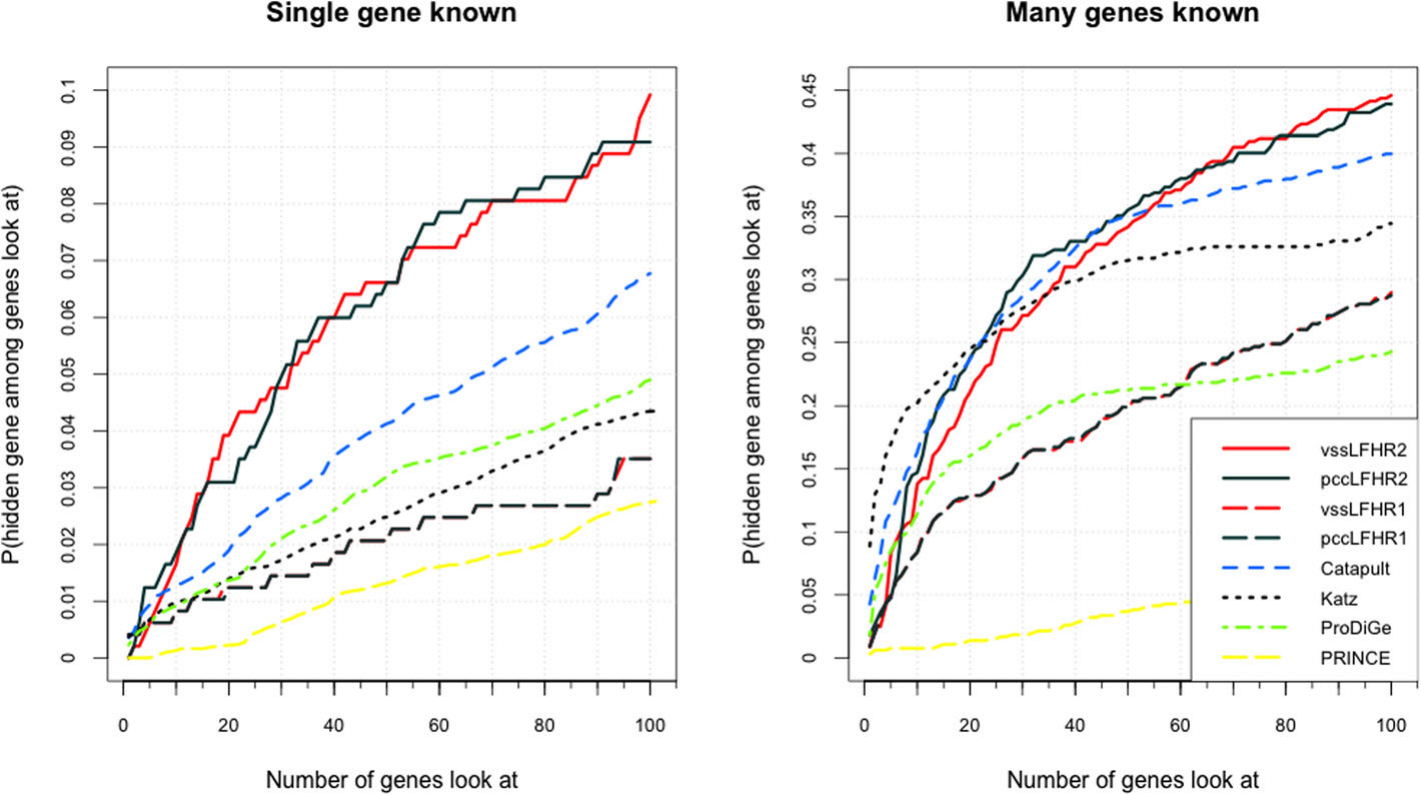
Comparison with state-of-art methods

We can see that in Fig. 3, we compare the results of model I and model II with the results of above four state-of-arts approaches. The vertical axis shows the probability that a true gene association is retrieved in the top-k (shown on the horizontal axis) predictions for given disease. In training set, human diseases on the dataset are divided into two parts. One part is associated with at least one human gene (many known genes), the other part is related to no known genes (single known gene). The dimensionality of latent factor vector is set as *D* = 10 and the parameters are set as *α*_*1*_ *= α*_*2*_ *=* 0.5, *β*_*1*_ *= β*_*2*_ *=* 0.001 for diseases with many known genes. In Fig. 3, we show the results of two kinds of diseases. The results of Model I (dashed black and dashed red) and model II (solid black and solid red) in PCFM is much better than other advanced approaches.

In Fig. 3, the result of model II is better than the result of Model I, this is likely caused by the lack of distinct groups in genes and diseases. Thus, G. Model II: Probability-based collaborative filtering model with personal regularization can perform better. For the two types of diseases, our method is 4% and 5% more efficient than above advanced methods.

### Effect of *a* and 훽

In the PCFM approach, *α* and *β* control the significances of gene–gene network and disease–disease network respectively. We set diverse *α* and *β* for two types of diseases by using model II. The dimensionality of latent factor vector is set as *D* = 5 for diseases with single known gene, and is set as *D* = 30 for diseases with many genes known.

**Fig. 4:**
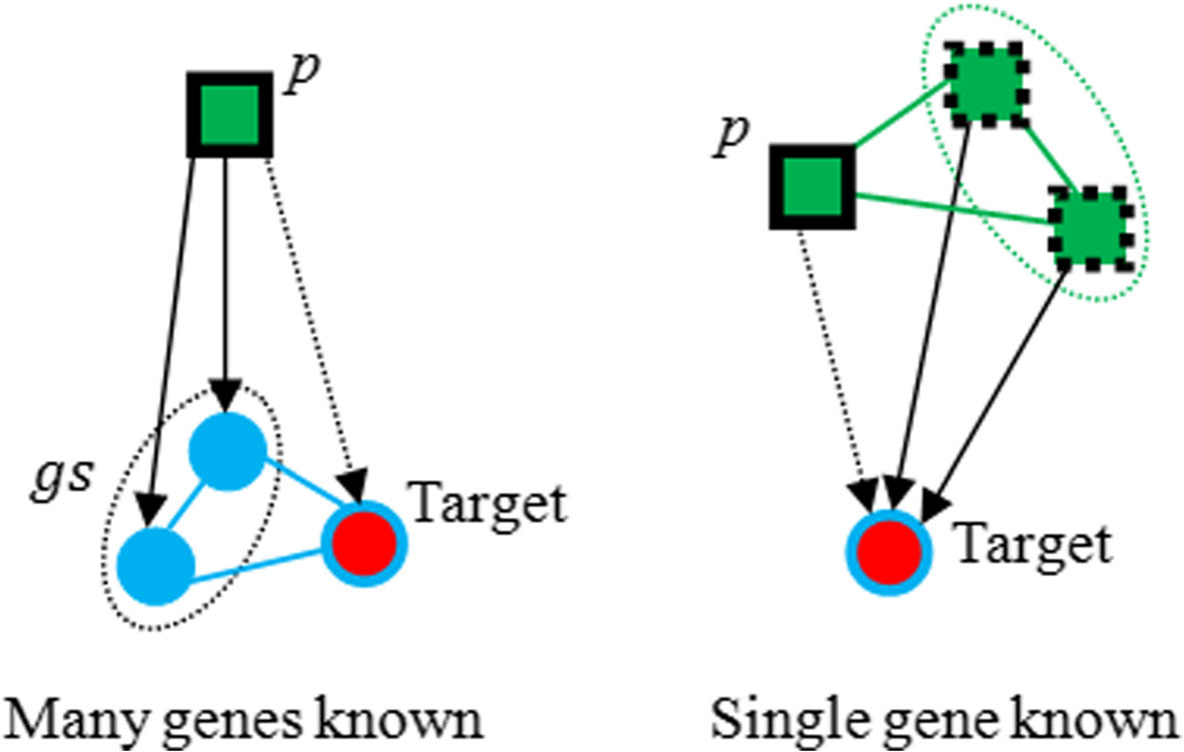
Training process of diseases with single gene known and with many genes known

We can see that in Table 1, model II performed better for diseases with many genes known than diseases with single gene known when *α* is bigger than *β*. And the result is opposite when *β* is smaller than α. If a disease *p* is related to certain genes *gs*(|*gs*| ≥ 1), the neighbor genes of *gs*, which are called Target in Fig. 4, are more likely to be related to disease *p*. Under the circumstances, disease–disease relationship network is less important than gene–gene relationship network, so bigger *α* cause better performance. Inversely, for diseases without any associations with human genes, it is tough to detect related genes. Thus, genes related to neighbor diseases of *p* is likely to build a association with *p*, and bigger 훽 will lead to a more outstanding performance.

**Table 1:** Effect of *α* and *β*

α		0.0001	0.001	0.005	0.01	0.05	0.1	0.5	1
β									
0.0001	S	0.043	0.048	0.029	0.035	0.035	0.023	0.027	0.027
	M	0.149	0.172	0.195	0.19	0.265	0.265	0.31	0.328
0.001	S	0.048	0.037	0.025	0.027	0.029	0.021	0.017	0.039
	M	0.186	0.154	0.197	0.183	0.308	0.344	*0.376*	0.369
0.005	S	0.037	0.033	0.023	0.029	0.014	0.014	0.01	0.027
	M	0.106	0.102	0.147	0.147	0.235	0.26	0.276	0.278
0.01	S	0.052	0.07	0.052	0.041	0.037	0.029	0.012	0.017
	M	0.093	0.077	0.07	0.054	0.136	0.197	0.133	0.163
0.05	S	0.089	0.118	0.107	0.083	0.066	0.052	0.068	0.045
	M	0.023	0.011	0.023	0.036	0.048	0.075	0.079	0.061
0.1	S	0.11	0.099	0.118	0.114	0.081	0.072	0.054	0.064
	M	0.063	0.045	0.023	0.059	0.029	0.045	0.027	0.032
0.5	S	*0.145*	0.107	0.128	0.076	0.099	0.091	0.072	0.066
	M	0.05	0.054	0.048	0.043	0.038	0.027	0.043	0.018
1	S	0.107	0.107	0.11	0.083	0.081	0.089	0.066	0.05
	M	0.068	0.068	0.059	0.045	0.045	0.052	0.063	0.048

The italicized value indicates the local optimal

**Fig. 5:**
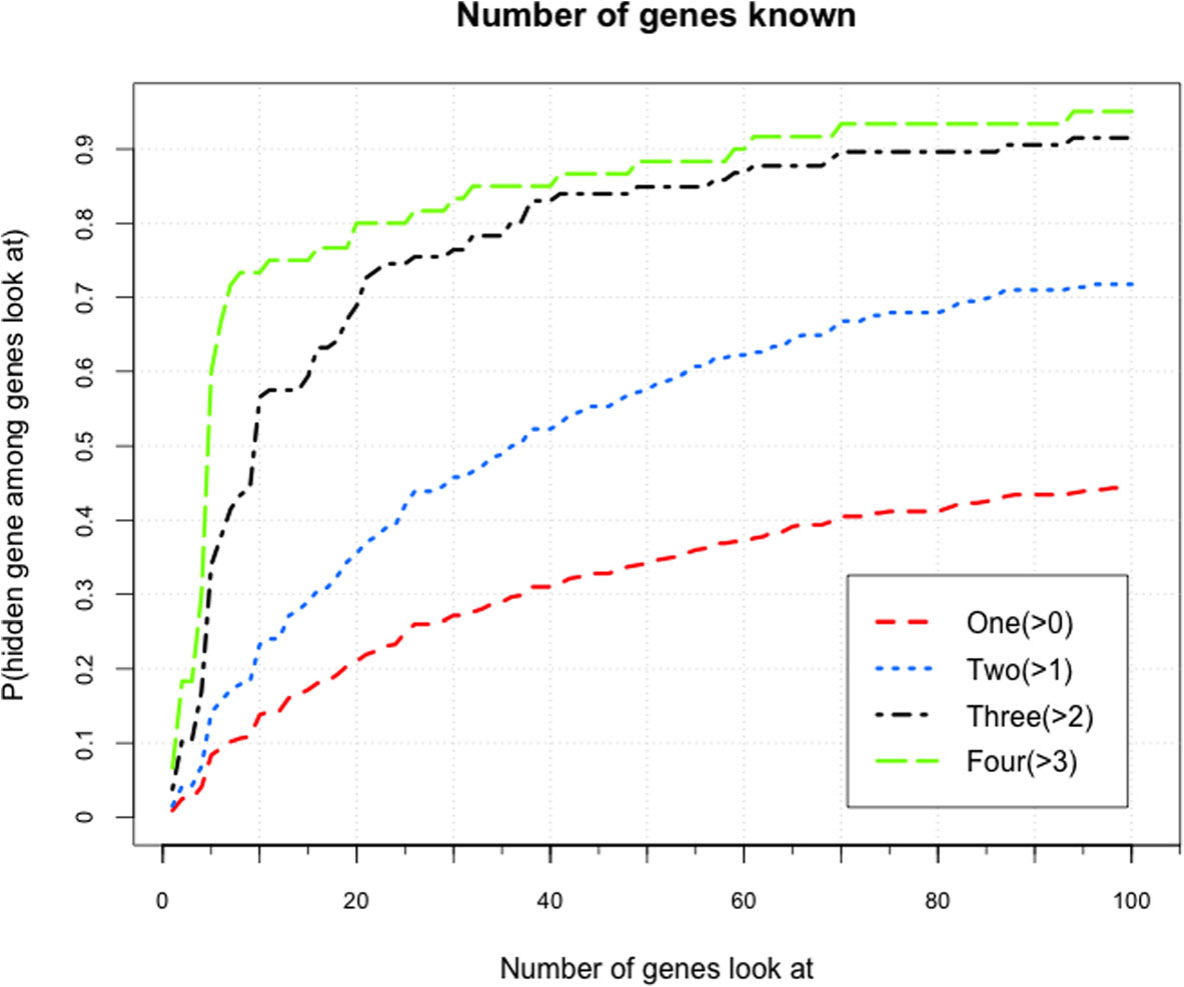
Accuracies of different numbers of genes known

It is assumed that the accuracy of PCFM would be enhanced if a human disease is related to more known genes. Thus, the dimensionality of latent factor vector is set as *D* = 60, and other parameters are set as *α*_*1*_ = *α*_*2*_ = 0.01, *β*_*1*_ = *β*_*2*_ = 0.5. We exploit Model II with VSS for prediction of diseases with many known genes. In Fig. 5, we show the performances of diseases with different numbers of several known genes. We can conclude that more associated known genes would generate a better performance. Particularly, when three or four genes are known to be related to a disease, the predicting accuracy rises extremely fast in the top 10 genes.

### Effect of dimensionality D of latent factor vector

**Fig. 6:**
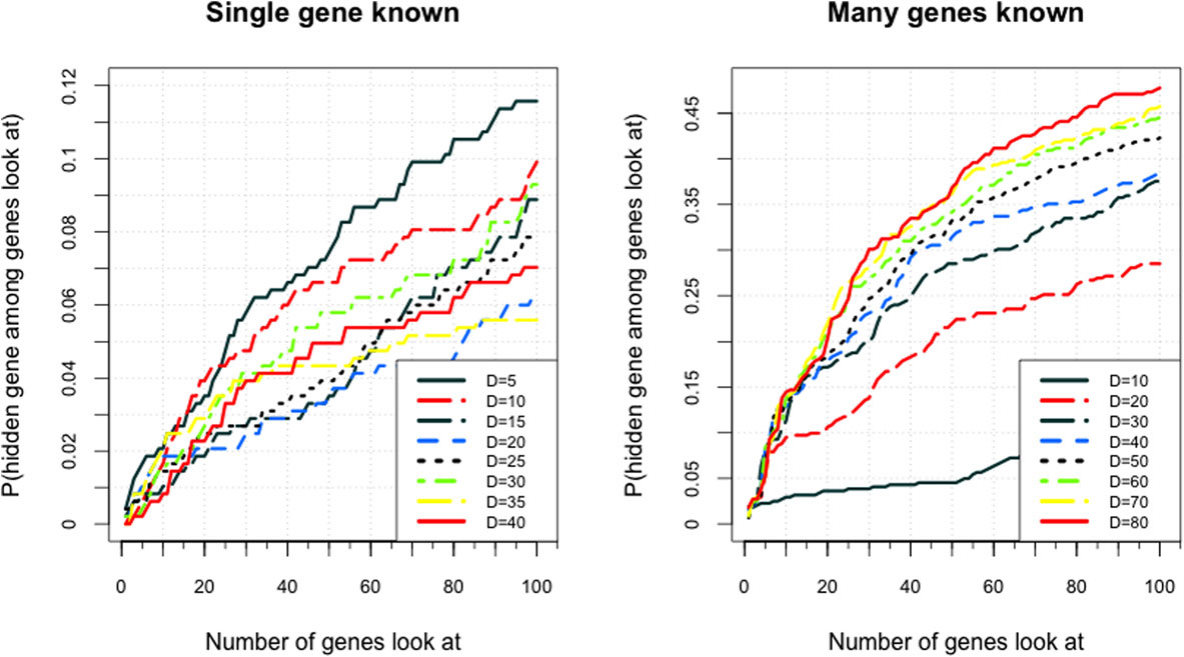
Accuracies of different dimensionalities of latent factor vector

The value of dimensionality of the latent factor vector can largely influence the accuracy of the experiment. If the dimensionality is very small, notwithstanding the complexity of calculation is reduced, the model may be underfitting. Thus, poor findings are obtained. By contrast, if the dimensionality is very large, more time will be spent for calculation, and overfitting of the model may result in poor performance. In our experiment, VSS is used to compute gene–gene similarities. For diseases with many known genes, we set *α*_*1*_ = *α*_*2*_ = *0.5* and *β*_*1*_ = *β*_*2*_ = *0.001*; for diseases with single known gene we set *α*_*1*_ = *α*_*2*_ = *0.05* and *β*_*1*_ = *β*_*2*_ = *0.5*. The experimental results are show in Fig. 6. Lower dimensionality is better than higher dimensionality for diseases with single known gene because of insufficient available training data. As such, the model is overfitting and the generalizability is reduced. For diseases with many known genes, the result improves when the dimensionality increases. At D = 80, PCFM is 7% better than Catapult in the top 100 genes. Nevertheless, more time is spent for calculation when dimensionality increases, whereas the accuracy improves gradually. Thus, we should set a suitable value of D to balance the time spent and precision.

### Discussion

With the research datasets related to genes and diseases increasing rapidly, a number of calculating strategies, like RWRH and CIPHER, have been developed for prediction of gene–disease relationships. Consequently, this proposed method should be further built up through research on theoretical prediction.

Our study is based on collaborative filtering model, and a probability conversion is defined. Utilizing PCFM, we detected gene–disease relationships, and it is regarded as a semi-supervised learning problem.

## Conclusion

Finding out gene–disease relationships is essential for understanding human disease mechanisms. As a result of the lack of negative samples, predicting gene–disease relationships is often regarded as a semi-supervised learning problem, which. Our PCFM approach was proposed for prediction of pathogenic human genes and for getting more precise consequence than other state-of-arts strategies. The problem is changed into a binary classification problem, with consideration that two vertexes would be alike if the Euclidean distance between these vertexes is short in a latent factor space. To leverage comprehensive prior information and get more accurate result, probability conversion is defined in this approach. In this experiment, it is proved that the proposed model is feasible. Accordingly, we can apply PCFM to enhance the efficiency of prediction markedly. In future research, more data resources including gene expression data may be utilized to establish the human gene network and to enhance the precision of prediction.
